# Cytokine and eicosanoid regulation by Schistosoma mansoniduring LSE penetration

**DOI:** 10.1155/S0962935193000109

**Published:** 1993

**Authors:** A. C. Fusco, B. Salafsky, T. Shibuya

**Affiliations:** 1 University of Illinois College of Medicine at Rockford Department of Biomedical Sciences 1601 Parkview Avenue Rockford Illinois 61107 USA; 2 Tokyo Medical College Department of Pharmacology Tokyo Japan

## Abstract

Cercarial penetration, in low to moderate numbers, does not cause a normal skin inflammatory response; therefore, the authors sought to determine whether cercariae can down-regulate keratinocyte activation and thus the secretion of pro-inflammatory cytokines and eicosanoids. Human living skin equivalent (LSE, Organogenesis) consisting of dermal, epidermal and stratum corneum-like layers was used as the skin substrate. The surface of the LSE membrane was exposed to 100 ng IFNγ or ~850 cercariae for 18 h. Incubation media and tissue was then assayed for IL-1α, IL-6, IL-8, TNFα, 5-HETE, 12-HETE, PGF_2_, LTB_4_, and LTC_4_ via RIA and Western Blots. TNFα was not detected. Secreted IL-1α levels were (mean ± S.E.M. (n)): Control, 1.03 ng ± 0.15 (11); IFNγ 1.90 ng ± 0.48 (5); cercariae, 1.79 ng ± 0.22 (22). In spite of this increase, cercariae down-regulated IL-8 (cercariae 11.13 ± 1.70 ng vs. IFNγ = 16.47 ± 0.29 ng, p = 0.04) and LTB_4_ (cercariae = 98.86 ± 19.65 pg/0.1 ml vs. IFNγ = 193.42 ± 44.21 pg/0.1 ml p = 0.02). No changes were seen in IL-6, 12-HETE, 5-HETE, and PGE_2_ levels. It is concluded that cercarial penetration causes a release of IL-1α consistent with skin trauma; however, schistosomulae may regulate the production of chemotactic (neutrophils, macrophages, T-cells, etc.) and activation factors such as IL-8 and LTB_4_.


Mediators of Inflammation, 2, 73–77 (1993)

### Cytokine and eicosanoid regulation by *Schistosoma mansoni* during LSE penetration

A. C. Fusco,[1,CA] B. Salafsky[1] and T. Shibuya[2]

[1]University of Illinois College of Medicine at Rockford, Department of Biomedical Sciences, 1601 Parkview Avenue, Rockford, Illinois 61107, USA; [2]Tokyo Medical College, Department of Pharmacology, Tokyo, Japan

[CA] Corresponding Author

**CERCARIAL penetration, in low to moderate numbers, does not cause a normal skin inflammatory response; therefore, the authors sought to determine whether cercariae can down-regulate keratinocyte activation and thus the secretion of pro-inflammatory cytokines and eicosanoids. Human living skin equivalent (LSE, Organogenesis) consisting of dermal, epidermal and stratum corneum-like layers was used as the skin substrate. The surface of the LSE membrane was exposed to 100 ng IFNγ or ~850 cercariae for 18 h. Incubation media and tissue was then assayed for IL-1α, IL-6, IL-8, TNFα, 5-HETE, 12-HETE, $PGF_2$, $LTB_4$, and $LTC_4$ *via* RIA and Western Blots. TNFα was not detected. Secreted IL-1α levels were (mean ± S.E.M. (*n*)): Control, 1.03 ng ± 0.15 (11); IFNγ 1.90 ng ± 0.48 (5); cercariae, 1.79 ng ± 0.22 (22). In spite of this increase, cercariae down-regulated IL-8 (cercariae = 11.13 ± 1.70 ng *vs.* IFNγ = 16.47 ± 0.29 ng, $p = 0.04$) and $LTB_4$ (cercariae = 98.86 ± 19.65 pg/0.1 ml *vs.* IFNγ = 193.42 ± 44.21 pg/0.1 ml $p = 0.02$). No changes were seen in IL-6, 12-HETE, 5-HETE, and $PGE_2$ levels. It is concluded that cercarial penetration causes a release of IL-1α consistent with skin trauma; however, schistosomulae may regulate the production of chemotactic (neutrophils, macrophages, T-cells, etc.) and activation factors such as IL-8 and $LTB_4$.**

**Key words:** Cercariae, Cytokines, Eicosanoids, Living Skin Equivalent, Penetration, *Schistosoma mansoni*

### Introduction

*Schistosoma mansoni* is primarily a tropical and semi-tropical parasitic disease which infects an estimated 250–300 million people. Infection occurs when a human host comes into contact with water containing the infective cercarial stage. Cercariae penetrate the skin barrier and undergo a series of biochemical, physiological and morphological changes resulting in the schistosomular stage. Schistosomulae remain in the skin for 24–48 h before migrating to the capillaries and eventually becoming established as adults in the hepatoportal system. Skin penetration by low to moderate (<200) numbers of *S. mansoni* cercariae does not cause a normal inflammatory response in man or most laboratory models (mice, hamsters).[1–7] This lack of inflammation is remarkable for two reasons: (1) It is known that the epidermis, especially the keratinocyte, is extremely active in immunoregulation and that the first manifestation of any trauma to the skin barrier is an inflammatory response.[8,9] (2) Cercariae and schistosomulae, *in vitro*, secrete a wide variety of eicosanoids which are potent inflammatory agents.[10–12] Since cytokine production, most notably IL-1 and TNFα, and eicosanoid synthesis are important mediators of inflammation, the authors sought to determine whether cercariae or schistosomulae control skin inflammation by regulating their production. In order to study these interactions, the Living Skin Equivalent (LSE) manufactured by Organogenesis was used. The LSE consists of a dermal layer containing human dermal fibroblasts embedded in a collagen matrix, an epidermal layer consisting of human keratinocytes in various states of differentiation including a fully developed stratum corneum, and a basement membrane-like layer separating the dermis and epidermis.

### Materials and Methods

*Animals: Biomphalaria glabrata* snails infected with *Schistosoma mansoni* were obtained from Dr Yung-san Liang, University of Lowell Center for Tropical Diseases. Infected snails were kept under the ideal population densities as stated by Coles.[13] Snails were fed alternately TetraMin® Fish food and Romaine lettuce. In addition, low grade chalk was put into each tank as a source of calcium.

*Tissue culture and host–parasite incubations:* LSE was purchased from Organogenesis, Inc. (Cambridge, MA) as TESTSKIN® and was maintained in the laboratory 1 week before use according to the manufacturer's directions (35°C, 5–8% $CO_2$).

© 1993 Rapid Communications of Oxford Ltd

Organogenesis Maintenance Media was supplemented with 3 $\mu$M arachidonic and linoleic acids. On the day of experimentation an Assay Ring (Organogenesis) was placed over the LSE tissue and sealed to the surface with sterile DuPont Silicone Sealant. The surface of the LSE (internal area of the assay ring) was then coated with 4 $\mu g/cm^2$ linoleic acid. The assay chambers were then placed in the Organogenesis Assay Tray (essentially a six-well tissue culture plate) containing 1.5 ml of Assay Media (1:1 mixture of low calcium Dulbecco's Modified Eagles Medium and Hams F-12 medium containing phenol red, 1.85 g/ml of sodium bicarbonate and 50 $\mu$g/ml gentamicin sulphate) and stabilized in this medium for 1 h. At the end of this time the Assay Media was changed and either approximately 850 cercariae in 200 $\mu$l aged tap water, or 100 ng IFN$\gamma$ were placed in the centre of the assay ring. Control assays contained only 4 $\mu g/cm^2$ linoleate. Incubation was for 18 to 20 h at 35°C and 6–8% $CO_2$. At the end of the incubation period, the media in each assay well was divided into three 0.5 ml samples and frozen at −80°C. The tissue was homogenized in 5 ml of assay media, divided into three aliquots, and frozen at −80°C.

*Western Blots:* Initial screening for cytokines was done by Western Blot and then quantitated by RIA. Samples were separated using the SDS-PAGE system described by Laemmli with minimal changes.[14] Optimal conditions were a 13% acrylamide gel (0.15 M Tris/HCl pH 8.8, 0.1% SDS) overlaid with a 4% acrylamide stacking gel (0.05 M Tris/HCl pH 6, 0.1% SDS) cast using 140 mm × 160 mm × 1.5 mm plates. Typical running conditions for two 1.5 mm thick gels were 6 h at 60 mA constant current after an initial current of 40 mA for 1 h. Western Blots were done on nitrocellulose membranes (BA-S NC, 0.2 $\mu$m pore size, Schleicher & Schuell, NH) using the Towbin buffer system (25 mM Tris, 192 mM glycine, 20% methanol, pH 8.3) in a Trans-Blot Electrophoresis Transfer Cell (BioRad, CA) at 30 V constant voltage overnight. The membrane was blocked with 3% gelatin in 20 mM Tris and 500 mM NaCl. The first antibody solution was 20 mM Tris, 500 mM NaCl, 0.05% Tween 20, 1% gelatin containing the following antibodies at a concentration of 1 mg/100 ml: rabbit anti-human IL-1$\alpha$ (Endogen P-420A), rabbit anti-human IL-8 (Endogen P-801), rabbit anti-human TNF$\alpha$ (Endogen P-300A), rabbit anti-human IL-6 (Endogen P-620). Incubation in the first antibody solution was for 2 h at room temperature. The second antibody solution contained the same buffer as the first with a 1:3 000 dilution of goat anti-rabbit IgG (H + L) alkaline phosphatase (BioRad). Incubation was for 1 h at room temperature. Colour was developed using an NBT/DCIP system as supplied in the BioRad Immuno-Blot Assay Kit. This system was easily able to separate and detect purified standards of IL-1$\alpha$ (pro ~31 KDa, expressed 17.5 KDa), IL-6 (pro 26 KDa, expressed 20.5 KDa), IL-8 (8.5 KDa), and TNF$\alpha$ (17 KDa).

*Radioimmunoassays:* RIAs were done using commercially available kits according to the manufacturer's instructions. Interleukin-1$\alpha$ [$^{125}$I], Interleukin-6 [$^{125}$I], Interleukin-8 [$^{125}$I], and TNF$\alpha$ [$^{125}$I] assay systems were purchased from Amersham (Arlington Heights, IL). All eicosanoid kits were tritiated and purchased from Advanced Magnetics (Cambridge, MA).

*Statistics:* Statistics were calculated using Microsoft Excel version 4.0 using the *t* test: two-sample assuming equal variances function. Significance was calculated at the $\alpha = 0.05$ level.

### Results

*Cercarial penetration of LSE:* Cercarial penetration into LSE membranes averaged 81 ± 2.6% as determined by recovery of cercariae from the membrane surface.

*Regulation of cytokine production:* Initial screening by Western Blots showed the presence of IL-1 (31 KDa form), IL-6 and IL-8 in all samples; however, the Western Blot did not prove sensitive enough to determine quantitative differences between these three cytokines. TNF$\alpha$ was not detected by Western Blot. Cytokine quantitation by RIA is shown in Fig. 1 (secreted) and Fig. 2 (tissue). These results show that cercarial penetration of LSE and topical treatment with IFN$\gamma$ caused an increase in IL-1$\alpha$ secretion over controls (controls = 1.03 ± 0.15 ng *vs.* IFN$\gamma$ = 1.90 ± 0.48 ng, $p = 0.02$; and cercariae = 1.79 ± 0.22 ng, $p = 0.02$). There was no significant difference in IL-1$\alpha$ secretion between IFN$\gamma$ treatment and cercarial penetration. IL-6 production was not significantly different between all three groups (average of 1.97 ± 0.35 ng). Cercariae down-regulated the production of IL-8 when compared to IFN$\gamma$ (cercariae = 11.13 ± 1.70 ng *vs.* IFN$\gamma$ = 16.47 ± 0.29 ng, $p = 0.04$). IL-8 levels in LSE exposed to cercariae were not significantly different from untreated controls (cercariae = 11.13 ± 1.70 ng *vs.* controls = 8.48 ± 1.47 ng, $p = 0.12$). Tissue cytokine levels remained relatively constant regardless of treatment. TNF$\alpha$ was not detected by RIA, confirming the Western Blot analysis.

*Regulation of eicosanoid production:* Figure 3 shows the results of cercarial regulation of eicosanoid

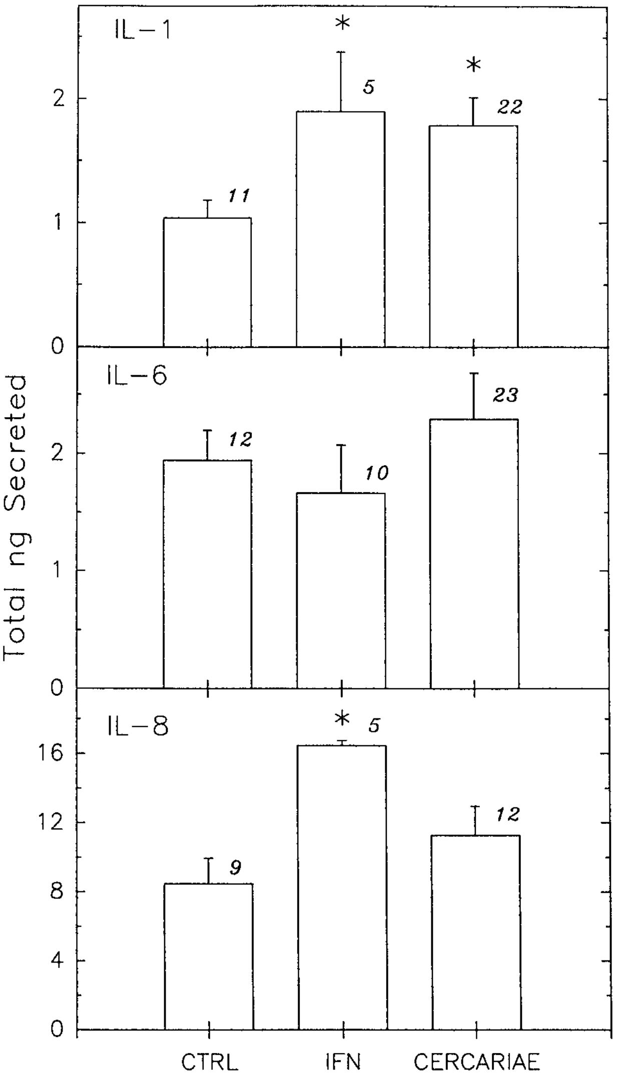

FIG. 1. Living Skin Equivalent (LSE, Organogenesis) secretion of cytokines into the assay media. The air interface side of the LSE (stratum corneum side) was coated with 4 $\mu g/cm^2$ linoleic acid and then exposed to either 100 ng IFN$\gamma$ (IFN), 850 *S. mansoni* cercariae (CER), or nothing (CTRL) for 18–20 h at 35°C. Cytokine levels in the assay media were measured directly by RIA. Numbers represent the number of replications for each group. Error bars are mean ± S.E.M. $*p < 0.05$ as compared with controls. Students *t* test. Nonspecific binding (assay media only) has been subtracted.

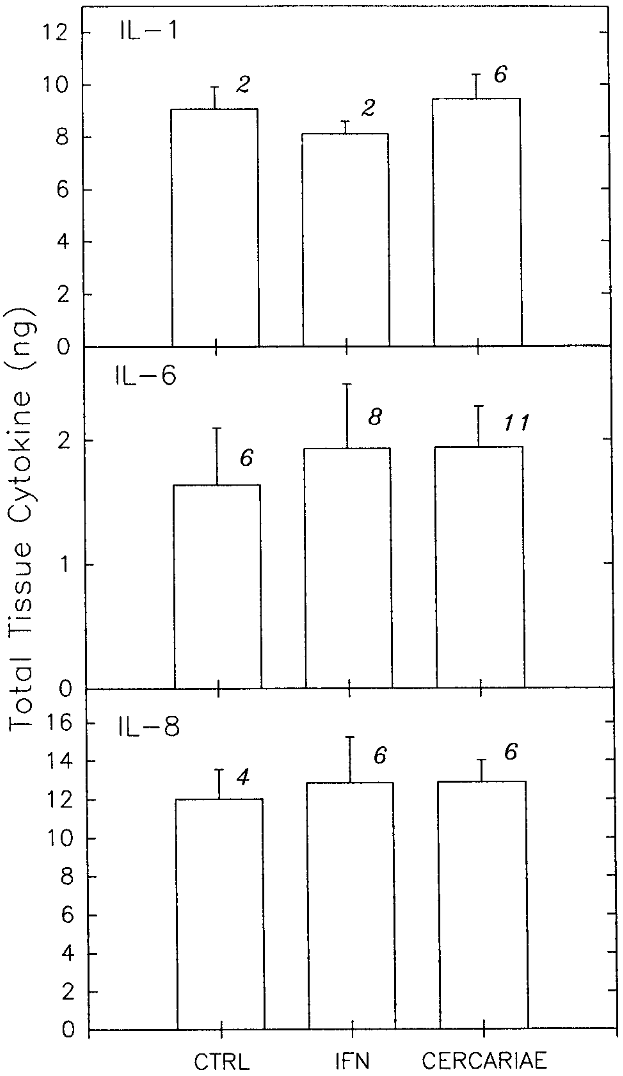

FIG. 2. Tissue cytokine levels in Living Skin Equivalent (LSE, Organogenesis) exposed to either 100 ng IFN$\gamma$, 850 *S. mansoni* cercariae (CER), or nothing (CTRL). Details as in Fig. 1 except that LSE tissue was homogenized in 5 ml of assay media and cytokines levels of the homogenate were measured by RIA. Numbers represent the number of replications for each group. Error bars are mean ± S.E.M. $*p < 0.05$ as compared with controls. Students *t* test. Nonspecific binding (assay media only) has been subtracted.

production. IFN$\gamma$ was used as a control inflammatory agent. IFN$\gamma$ treatment caused an increase in $LTB_4$ of almost 260% although no significant increase was seen in the other eicosanoids examined. Cercarial penetration caused a decrease in $LTB_4$ production when compared with the increase caused by IFN$\gamma$ (control = 73.9 ± 30.42 pg/0.1 ml *vs.* IFN$\gamma$ = 193.42 ± 44.21 pg/0.1 ml *vs.* cercariae = 98.86 ± 19.65 pg/0.1 ml). There were no statistically significant changes in 12-HETE, 5-HETE, $PGE_2$, and $LTC_4$ production in LSE exposed to either IFN$\gamma$ or cercariae.

### Discussion

The skin is a formidable barrier against infection and it is now known that the keratinocyte plays an active and key role in skin immunology. Keratinocytes are normally found in a resting state. In this state keratinocytes contain internal stores of IL-1, relatively few IL-1 receptors, and lack the enzymes necessary to convert pro IL-1$\beta$ to its active form.[15–18] In addition, resting keratinocytes also secrete an IL-1r antagonist (IL-1ra) to further prevent response to IL-1 under normal (resting) conditions.[19] As keratinocytes differentiate into the stratum corneum, the IL-1 still remains associated with the cell. Both the stratum corneum and sweat have been shown to contain high levels of IL-1$\alpha$ and IL-1$\beta$.[20,21] When the stratum corneum layer is disrupted and/or keratinocytes are damaged this causes a release of proinflammatory IL-1. IL-1$\alpha$ (pro or processed) then binds to receptors contained on nearby keratinocytes. This initiates the up-

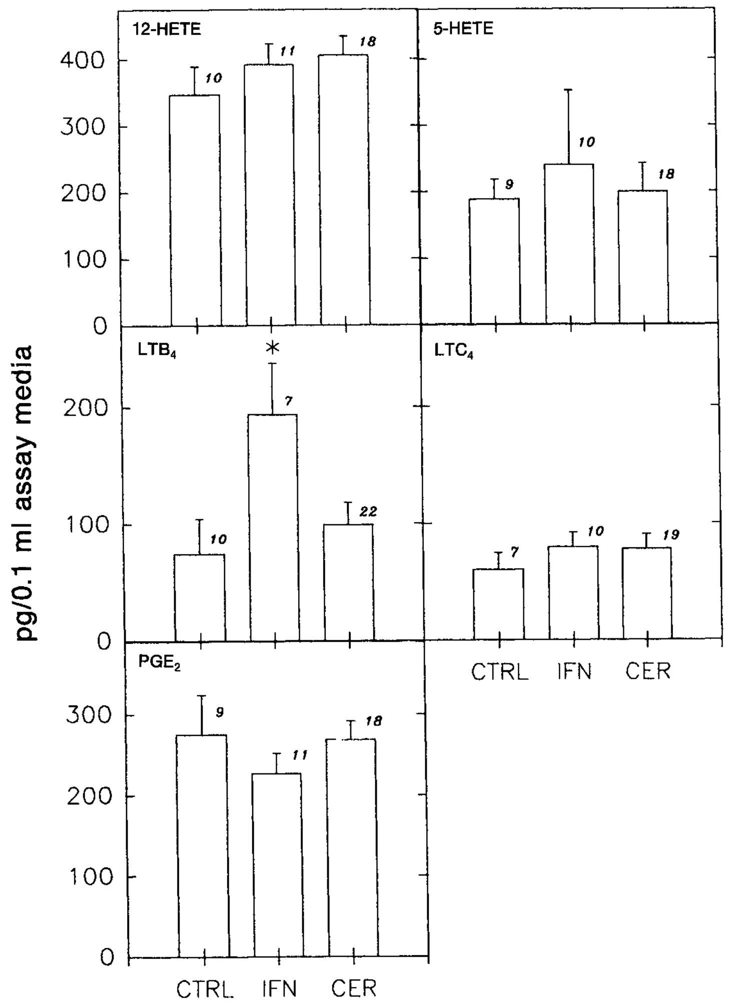

FIG. 3. Living Skin Equivalent (LSE, Organogenesis) secretion of eicosanoids into the assay media. Details are as in Fig. 1. Eicosanoid levels in the assay media were measured directly by RIA. Numbers represent the number of replications for each group. Error bars are mean ± S.E.M. $^*p < 0.05$ as compared to controls. Students $t$ test. Nonspecific binding (assay media only) has been subtracted.

regulation of IL-1 receptors (IL-1r), the presumed down-regulation of IL-1ra, and the synthesis and secretion of additional IL-1 by neighbouring keratinocytes. The keratinocyte is now considered 'activated'. Keratinocytes can also be activated by PGE (increase IL-1r), $LTB_4$ (induces secretion of IL-1) or IFN$\gamma$ (induces both IL-1 secretion and increase in IL-1r).

Once keratinocytes are 'activated' they synthesize and secrete a wide variety of cytokine and eicosanoid factors involved in inflammation. These factors include those chemotactic for various inflammation cells (IL-8: neutrophils, macrophages, T-cells; 12-(R)HETE: neutrophils; $LTB_4$: neutrophils, eosinophils; 5-HETE: eosinophils, neutrophils, fibroblasts; PAF: eosinophils; IL-1: leukocytes; and TNF$\alpha$: leukocytes), factors that cause inflammatory cell activation (IL-6: T-cell; GM-CSF: neutrophils, eosinophils; IL-8: neutrophils; IL-1, G-CSF, and TNF$\alpha$: neutrophils, eosinophils, macrophages) and factors that cause the proliferation of fibroblasts and keratinocytes and are instrumental in the wound healing process (12-(R)HETE, 5-(S)HETE, $LTB_4$, IL-1, and TGF).

Given this sequence of events, cercarial penetration should cause an intense inflammatory reaction. During skin penetration the stratum corneum should be disrupted thereby releasing stored IL-1. In addition, penetration and migration of *S. mansoni* causes extensive tissue destruction,[4–6] thus further IL-1 should be released. Finally, cercariae and schistosomulae synthesize both $PGE_2$ and $LTB_4$, *in vitro*, both of which should activate keratinocytes. To further complicate the matter, schistosomulae have been shown to be killed, *in vitro*, by macrophages activated by IFN$\gamma$ and TNF,[22,23] and platelets stimulated by TNF.[24] Also, the schistosomulae itself is a source of foreign antigen and should activate the skin SALT (skin associated lymphoid tissue) system (predominately Langerhans's cells in humans) which, again, should induce an inflammatory response resulting in schistosomular destruction. Yet, while most EM and light microscopic studies of cercarial penetration have shown tissue destruction along the path of schistosomular migration, they have also shown a remarkable absence of cell types associated with the inflammatory response. Even more puzzling, schistosomulae remain at the junction of the epidermis and dermis for 24 to 48 h before transversing the basement membrane and entering the dermis. During this entire time, a typical inflammatory response does not occur. In fact, several autoradiographic studies have shown that the skin is NOT a major site for schistosomular attrition[25–27] even after vaccination by irradiated cercariae.[28] The main advantage of using LSE is that cercarial–keratinocyte interactions can be investigated apart from the SALT system and inflammatory cell infiltration. Unfortunately, this is also a major drawback since these important systems cannot be studied. Still, the knowledge gained using simpler *in vitro* systems, such as the LSE, can be used to gain insight into more complex systems.

The results presented here show that cercarial penetration does indeed cause an increase in the secretion of IL-1 (pro-form by Western Blot) similar to that caused by IFN$\gamma$ treatment. This is consistent with Kupper's hypothesis.[8] Skin trauma should cause a release of IL-1 from damaged keratinocytes. However, we also see that schistosomulae appear to regulate the synthesis of IL-8 and $LTB_4$, thus (potentially) blunting some of the effects of the increased IL-1 levels. Moreover, many other inflammatory components are not increased over control levels. Clearly, keratinocytes are not responding normally to increased IL-1 levels. The authors propose that schistosomulae may be regulating the skin immune system by down-regulating inflammatory components leading to cellular infiltration. This may be done by either synthesizing or causing the synthesis of an IL-1ra-like or contra-IL-1 like molecule.

### References

1. Cort WW. Studies on schistosome dermatitis. XI. Status of knowledge after more than twenty years. *Am J Hyg* 1950; **52(3)**: 251–307.
2. Cort WW. Studies on schistosome dermatitis. I. Present status of the subject. *Am J Hyg* 1936; **23**: 349–371.
3. Bruce JI, Pezzlo F, McCarty JE, Yajima Y. Migration of *Schistosoma mansoni* through mouse tissue. Ultrastructure of host tissue and integument of migrating larva following cercarial penetration. *Am J Trop Med Hyg* 1970; **19(6)**: 959–981.
4. Wheater PR, Wilson RA. *Schistosoma mansoni:* a histological study of migration in the laboratory mouse. *Parasitology* 1979; **79**: 49–62.
5. Rifkin E. Interaction between *Schistosoma mansoni* schistosomules and penetrated mouse skin at the ultrastructural level. In: Cheng TC, ed. *The Biology of Symbiosis*, Baltimore: University Park Press, 1971; 25–43.
6. Stirewalt MA, Dorsey CH. *Schistosoma mansoni:* Cercarial penetration of host epidermis at the ultrastructural level. *Exp Parasitol* 1974; **35**: 1–14.
7. Crabtree JE, Wilson RA. *Schistosoma mansoni:* An ultrastructural examination of skin migration in the hamster cheek pouch. *Parasitology* 1985; **91**: 111–120.
8. Kupper TS. Role of epidermal cytokines. In: Oppenheim JJ, Shevach EM, eds. *Immunophysiology. The role of cells and cytokines in immunity and inflammation*, New York: Oxford University Press, 1990; 285–305.
9. Kupper TS. Immune and inflammatory processes in cutaneous tissues. Mechanisms and speculations. *J Clin Invest* 1990; **86**: 1783–1789.
10. Salafsky B, Fusco AC. *Schistosoma mansoni:* A comparison of secreted vs nonsecreted eicosanoids in developing schistosomulae and adults. *Exp Parasitol* 1987; **64**: 361–367.
11. Fusco AC, Salafsky B, Kevin M. *Schistosoma mansoni:* eicosanoid production by cercariae. *Exp Parasitol* 1985; **59**: 44–50.
12. Fusco AC, Salafsky B, Whitely K, Yohe S. *Schistosoma mansoni:* pH dependence of cercarial eicosanoid production, penetration, and transformation. *Exp Parasitol* 1987; **64**: 139–146.
13. Coles GC. The effect of diet and crowding on the shedding of *Schistosoma mansoni* cercariae by *Biomphalaria glabrata. Ann Trop Med Parasitol* 1973; **67**: 419–423.
14. Laemmli UK. Cleavage of structural proteins during the assembly of the head of bacteriophage T4. *Nature* 1970; **227**: 680–685.
15. Kupper TS. Production of cytokines by epithelial tissues. A new model for cutaneous inflammation. *Am J Dermatopath* 1989; **11(1)**: 69–73.
16. Kupper TS, Chua AO, Flood P, McGuire J, Gubler U. Interleukin 1 gene expression in cultured human keratinocytes is augmented by ultraviolet irradiation. *J Clin Invest* 1987; **80**: 430–436.
17. Kupper TS. Interleukin 1 and other human keratinocyte cytokines: molecular and functional characterization. *Adv Dermatol* 1988; **3**: 293–301.
18. Hauser C, Saurat JH, Schmitt A, Jaunin F, Dayer JM. Interleukin 1 is present in normal human epidermis. *J Immunol* 1986; **136**: 3317–3323.
19. Hammerberg C, Arend WP, Fisher GF, *et al.* Human epidermis constitutively express the IL-1 receptor antagonist. *FASEB J* 1991; **5(4)**: A544. (Abstract)
20. Gahring LC, Buckley A, Daynes RA. Presence of epidermal-derived thymocyte activating factor/interleukin-1 in normal human stratum corneum. *J Clin Invest* 1985; **76**: 1585–1591.
21. Didierjean L, Gruaz D, Dayer JM, Saura JH. Human eccrine clean sweat contains high amounts of interleukin alpha and beta. *J Invest Dermatol* 1989; **92(3)**: 420. (Abstract)
22. Esparza I, Mannel D, Ruppel A, Falk W, Krammer PH. Interferon gamma and lymphotoxin or tumor necrosis factor act synergistically to induce macrophage killing of tumor cells and schistosomular of *Schistosoma mansoni. J Exp Med* 1987; **166**: 589–594.
23. Kubelka CF, Ruppel A, Krammer PH, Gemsa D. Killing of schistosomula of *Schistosoma mansoni* by macrophages: induction by T-cell clone-derived lymphokines and interferon-gamma. *Parasitol* 1986; **92**: 325–336.
24. Damonneville M, Wietzerbin J, Pancre V, *et al.* Recombinant tumor necrosis factors mediate platelet cytotoxicity of *Schistosoma mansoni* larvae. *J Immunol* 1988; **149(11)**: 3962–3965.
25. Mangold BL, Dean DA. Autoradiographic analysis of *Schistosoma mansoni* migration from skin to lungs in naive mice. Evidence that most attrition occurs after the skin phase. *Am J Trop Med Hyg* 1983; **32**: 785–789.
26. Dean DA, Mangold BL. Autoradiographic analysis of resistance to reinfection with *Schistosoma mansoni* in mice. Evidence that the liver is a major site of worm elimination. *Am J Trop Med Hyg* 1984; **33**: 97–103.
27. Georgi JR. *Schistosoma mansoni:* quantification of skin penetration and early migration by differential external radioassay and autoradiography. *Parasitology* 1982; **84**: 263–281.
28. Dean DA, Mangold BL, Georgi JR, Jacobson RH. Comparison of *Schistosoma mansoni* migration patterns in normal and irradiated cercaria-immunized mice by means of autoradiographic analysis. Evidence that worm elimination occurs after the skin phase in immunized mice. *Am J Trop Med Hyg* 1984; **33**: 89–96.

**Received 10 November 1992; accepted in revised form 8 December 1992**
